# Ruptured sinus of Valsalva aneurysm mimicking infective endocarditis in a patient with Down syndrome: case report

**DOI:** 10.1093/ehjcr/ytag495

**Published:** 2026-07-09

**Authors:** Jan Harpula, Julia Sekuła, Paweł Żurek, Wojciech Wojakowski, Ewa Peszek-Przybyła

**Affiliations:** Department of Cardiology and Structural Heart Diseases, Medical University of Silesia, Ziolowa 45 Street, Katowice 40-514, Poland; Department of Cardiology and Structural Heart Diseases, Medical University of Silesia, Ziolowa 45 Street, Katowice 40-514, Poland; Department of Cardiac Surgery, Medical University of Silesia, Ziolowa 45 Street, Katowice 40-514, Poland; Department of Cardiology and Structural Heart Diseases, Medical University of Silesia, Ziolowa 45 Street, Katowice 40-514, Poland; Department of Cardiology and Structural Heart Diseases, Medical University of Silesia, Ziolowa 45 Street, Katowice 40-514, Poland

**Keywords:** Down syndrome, Sinus of Valsalva rupture, Infective Endocarditis, Case report

## Abstract

**Background:**

Sinus of Valsalva aneurysm is a rare structural anomaly that could be congenital or acquired, usually silent until the moment of rupture. Its diagnosis can be challenging as the turbulent jet flow may mimic vegetations or abscesses associated with infective endocarditis (IE).

**Case summary:**

A 31-year-old woman with Down syndrome presented with signs of severe right heart failure and a history of recurrent fevers. Initial echocardiography revealed a massive left-to-right shunt and an echogenic structure in the aortic root, raising suspicion of a paravalvular abscess—patient was treated initially with intravenous antibiotics. However, due to negative blood cultures and imaging findings (coronary computed tomography and cardiac magnetic resonance), lesion was reclassified as a ruptured non-coronary sinus of Valsalva aneurysm (RSOV) fistulizing into the right atrium (Qp:Qs 3.5). Due to the ambiguity of the diagnosis and initial clinical stability, elective surgery was planned after antibiotics course was completed and heart failure treatment optimalization. One month later, the patient presented with acute heart failure event with distribution shock. Emergency surgery was performed. Intraoperative findings confirmed RSOV without signs of infection. The aneurysm was excised with fistula closure, and the aortic valve replaced. The patient made a full recovery.

**Discussion:**

This case highlights the diagnostic overlap between RSOV and IE. It underscores the critical role of multimodality imaging in excluding infection and defining anatomy. Furthermore, it demonstrates that in cases of massive left-to-right shunting, delaying surgery carries a high risk of rapid haemodynamic collapse.

Learning points

- Ruptured sinus of Valsalva aneurysm should be a differential diagnosis in patients presenting with acute right heart failure, which could appear like infective endocarditis.
- Multimodality imaging (coronary computed tomography angiography and cardiac magnetic resonance) is superior to echocardiography alone in differentiating mechanical complications from infectious processes (abscesses) and for precise surgical planning.

## Introduction

Aneurysm of the sinus of Valsalva is a rare cardiac anomaly, occurring in ∼0.09% of the general population.^1^ Its mechanism is a deficiency in the fusion of the aortic media and the annulus fibrous tissue. It is often congenital and associated with other connective tissue disorders or chromosomal abnormalities like Trisomy 21.^2^ Rupture of the aneurysm into a cardiac chamber creates an acute shunt, leading to heart failure. A major diagnostic challenge arises when the ruptured tissue or turbulent flow mimics the appearance of vegetations or abscesses, suggesting infective endocarditis (IE).^3^ We present a case of a young woman with Down syndrome where this diagnostic dilemma led to a conservative initial strategy, resulting in a life-threatening haemodynamic collapse.

## Summary figure

**Figure:**
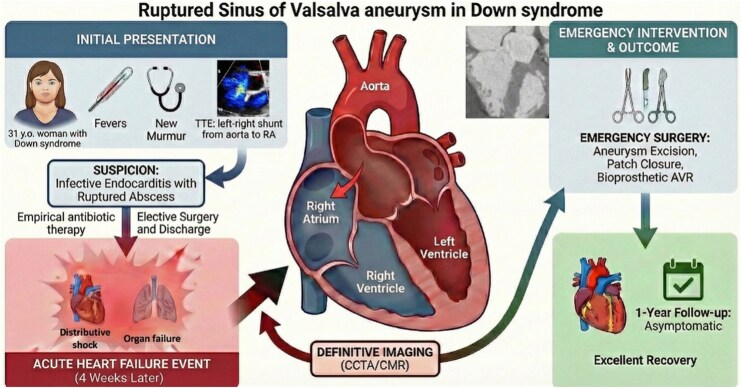


## Case presentation

A 31-year-old female with Down syndrome and hypothyroidism was referred to cardiology clinic due to progressive dyspnoea, abdominal distension, and lower limb oedema. Her medical history included recurrent fevers over the preceding weeks, which, combined with a new heart murmur, raised a high suspicion of IE. On admission, the patient was hemodynamically stable. Examination revealed jugular venous distension, hepatomegaly, ascites, and loud murmur with systolic accentuation at the left sternal border. Laboratory tests showed markedly elevated N-terminal pro-B-type natriuretic peptide (6326 pg/mL, N: 0–125 pg/mL) and mild anaemia (haemoglobin of 12 g/dL, N: 12–14.7 g/dL). Inflammatory markers were: [C-reactive protein16 mg/L (N: <5 mg/dL)], procalcitonin normal, white blood cells of 7.58 × 10^3^/µL (N: 3.9–9.5 × 10^3^/µL), and three sets of blood cultures remained negative. Baseline electrocardiogram did not show any significant changes (see Supplementary material online, *Figure S1*). The search for other, potentially inflammatory foci was performed; however, there were no signs of infection (gastrointestinal tract, urinary tract, oral cavity).

Transthoracic echocardiography (TTE) demonstrated normal left ventricular ejection function (60%) with right ventricular (RV) and right atrial (RA) dilatation (*Figure 1*). A turbulent flow was visualized between the aortic root and the RA. Transoesophageal echocardiography (TEE) revealed a cavity adjacent to the non-coronary aortic valve cusp, initially interpreted as a possible drained paravalvular abscess. To clarify the diagnosis, cardiac magnetic resonance (CMR, *Figure 2*) and coronary computed tomography angiography (CCTA, *Figure 3*; see Supplementary material online, *Figure S2*) were performed. Both excluded paravalvular abscess and confirmed a thin-walled, windsock-like aneurysm of the non-coronary sinus of Valsalva (18 × 16 mm) rupturing into the RA. A small patent foramen ovale (PFO) was also noted. In the CMR, the pulmonary-to-systemic flow ratio (Qp:Qs) was calculated at 3.5.

**Figure 1:**
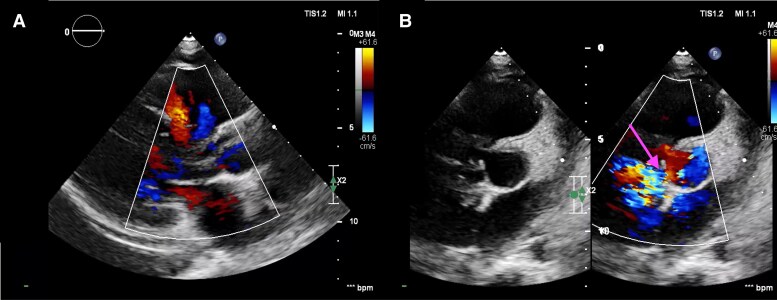
Transthoracic echocardiography on admission. (*A*) LAX (long axis) view and (*B*) modified SAX (short axis) view, pink arrow showing a turbulent flow between sinus of Valsalva and right heart.

**Figure 2:**
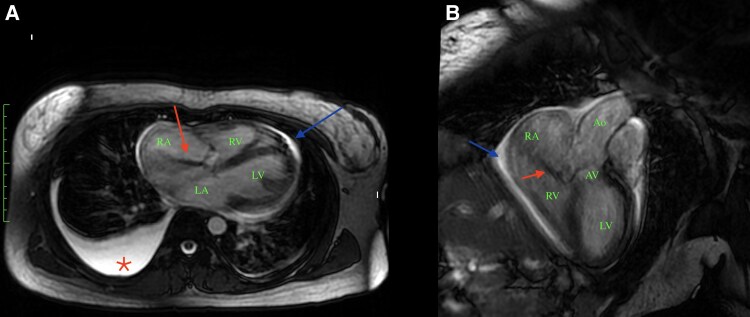
Cardiac magnetic resonance: (*A*) four-chamber cine view, (*B*) LVOT (left ventricular outflow tract) cine view. Red arrow pointing on the jet from right sinus of Valsalva to right atrium. Blue arrow pointing pericardial effusion. Red asterisk marking right pleural effusion.

**Figure 3:**
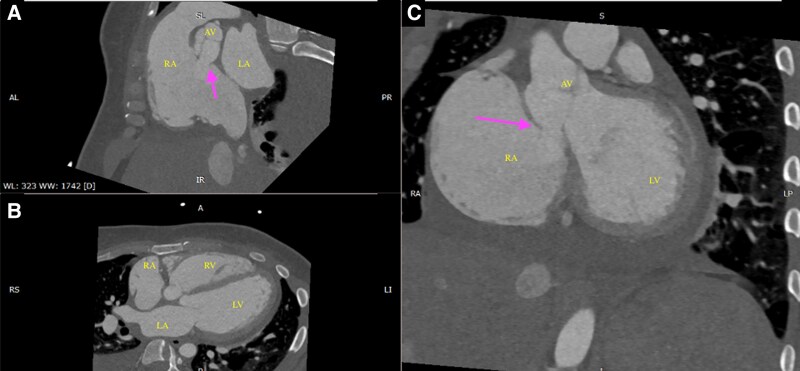
Coronary computed tomography angiography multi-planar reconstructions of the ruptured right sinus of Valsalva—pink arrow pointing on the rupture.

As the patient was initially under the suspicion of IE, empirical intravenous antibiotics therapy was started. Initially, the patient received 2 minor points (fever, predisposing heart condition) and 1 major clinical criterion (myocardial involvement—as initially lesion was addressed as abscess) in the Duke Criteria. However, with negative blood cultures but somewhat persistent diagnostic uncertainty regarding potential healed endocarditis, with given patient's clinical and haemodynamic stability, a Heart Team decision was made to complete the antibiotics therapy (4 × 3 g of ampicillin) and plan for semi-elective surgery 6 weeks apart [as the 2023 European Society of Cardiology (ESC) Guidelines for IE depicts that such patient could undergo surgery in non-urgent manner]—main rationale for such decision was to lower the surgical procedure risk as well as minimalization of potential valve infection. The patient was discharged home but readmitted 4 weeks later due to acute heart failure event. Upon second admission, she presented with signs of low cardiac output, severe hypotension, and oliguria. Echocardiography showed progression of RV inflow tract diameter (from initial 42 to 49 mm). Despite pharmacological support (intravenous furosemide followed by torsemide, potentialized with hydrochlorothiazide, dobutamine infusion), she developed cardiogenic and distributive shock with venous excess ultrasound assessment at Grade 3 with signs of multi-organ failure (acute kidney injury with creatinine 2.3 mg/dL, N: 0.51–0.95 mg/dL) and severe systemic congestion (*Figure 4*).

**Figure 4:**
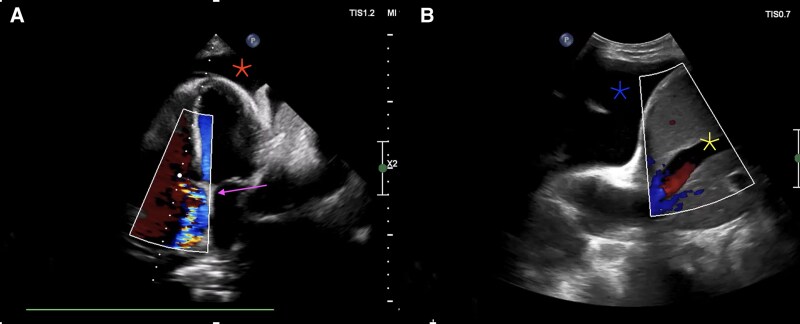
(*A*) Transthoracic echocardiography four-chamber view and (*B*) subcostal view (as part of FAST - focused assessment of sonography for trauma), during the acute heart failure event. Pericardial (red asterisk) and peritoneal (blue asterisk) effusion visible, with enlargement of hepatic veins (yellow asterisk) (venous excess ultrasound Grade 3). Large tricuspid regurgitation jet (pink arrow) visible.

As with the *de novo* poor haemodynamic tolerance, the patient was qualified (in accordance with the 2023 ESC IE Guidelines) and underwent emergency surgery. Via a standard sternotomy, cardiopulmonary bypass (CPB) was established utilizing ascending aortic and bicaval cannulation. The heart was arrested using Del Nido cardioplegia. Following a transverse aortotomy, the aortic valve was inspected and excised. A large, windsock-like aneurysm originating from the base of the non-coronary sinus was identified. There were no vegetations or signs of active inflammation.

The aneurysmal sac was excised, and the aortic defect was closed using a patch. The aortic valve was replaced with a 19-mm Inspiris Resilia bioprosthesis. Rationale for choosing a bioprosthesis rather that mechanical valve was mainly due to anticoagulation issue. As our patient presented with somewhat intellectual disability and required additional caregivers in everyday life, bioprosthesis seemed a much more suitable solution, without the need for strict international normalized ratio adherence. Subsequently, the PFO was directly closed. The patient was successfully weaned from CPB. Intraoperative TEE confirmed excellent function of the bioprosthesis with no residual left-to-right shunt. Chest closure was delayed due to coagulopathy requiring prolonged hemostasis.

The post-operative course was uneventful. Renal function recovered, and the patient was discharged home on Day 10 post-surgery. Follow-up imaging confirmed excellent prosthetic valve function and no residual shunt. Our patient is still under follow-up, showing no signs of bioprosthesis dysfunction (mild paravalvular leakage) or heart failure at 2 years post-surgery—on the last follow-up of TTE, the RV inflow diameter was 40.6 mm, with tricuspid annular plane systolic motion of 19 mm and RV S′ of 8 cm/s, with mild tricuspid regurgitation (*Figure 5*).

**Figure 5:**
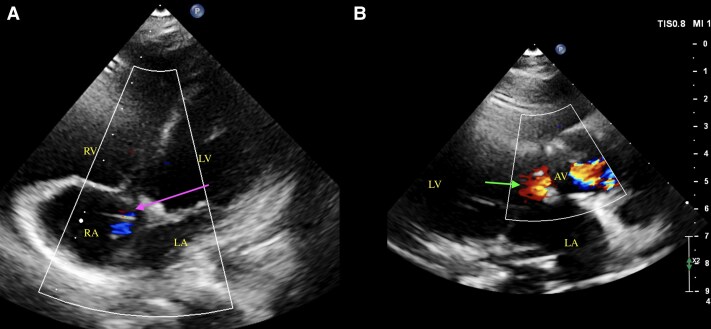
Follow-up transthoracic echocardiography (2 years after index procedure). (*A*) Four-chamber view, (*B*) LAX view. Pink arrow pointing on mild tricuspid regurgitation, and green arrow pointing on mild peri-valvular leak from aortic valve bioprosthesis.

## Discussion

Ruptured sinus of Valsalva aneurysm (RSOV) in patients with Down syndrome presents a set of challenges. The anatomy of the aortic root in Trisomy 21 may be prone to connective tissue deficiency, predisposing to aneurysm formation.^2^ The learning point in this case is the suspicion of endocarditis. The irregular edges of a ruptured aneurysm can easily be mistaken for vegetations on echo.^3,4^ In our case, the clinical presentation and history of fever were misleading. Multimodality imaging was the key to correcting the diagnosis. While TTE is the first-line tool, CCTA and CMR provide anatomical definition and should be mandatory in ambiguous cases. However, differentiation of a structural RSOV from an abscess or infective pseudoaneurysm heavily relies on specific features in multimodality imaging—demonstrating a smooth, thin-walled outpouching continuous with the aortic root, rather than the thick, irregular cavity typical of an abscess. Furthermore, the imaging lacked the hallmarks of active local infection: there was no surrounding inflammatory soft-tissue stranding, no peripheral rim enhancement, and no focal hypodensities or hypointensities indicating. These favoured a mechanical rupture over an active infective process, although the final confirmation was obtained intraoperatively.^5^ Finally, the decision regarding surgical timing is critical. While guidelines suggest urgent surgery for RSOV, ‘healed’ IE typically warrants a delay to reduce surgical risk. In this case, the massive volume overload (Qp:Qs 3.5) on a right ventricle likely led to rapid decompensation.^6^ This suggests that in the presence of such a significant shunt, mechanical correction should not be delayed.

## Supplementary Material

ytag495_Supplementary_Data

## Data Availability

The data underlying this article are available in the article and in its online Supplementary material.

## Lead author biography

A cardiac imaging enthusiast, with aprimary focus on echocardiography, cardiac computed tomography, nuclear imaging, and magnetic resonance imaging. His research interests also include the physiology of coronary circulation (Ischaemia and Angina without obstructive coronary artery disease) and sports cardiology.

## Notes

**Consent:** The authors confirm that consent for submission and publication of this case report including images and associated text has been obtained, in line with COPE guidance.

## References

[1] Feldman DN, Roman MJ. Aneurysms of the sinuses of Valsalva. Cardiology 2006;106:73–81. 16612073 10.1159/000092635

[2] Benatar A, Decraene T, Feenstra A. Ruptured sinus of Valsalva aneurysm in a child with down syndrome: a rare cardiac anomaly. Med Sci Monit 2010;16:CS135–C137. 20980963

[3] Bhimani SA, Challapudi G, Najm HK, Komarlu R. Ruptured sinus of Valsalva aneurysm mimicking infective endocarditis in DiGeorge syndrome. J Cardiovasc Echogr 2019;29:177–179. 32090000 10.4103/jcecho.jcecho_37_19 PMC7011494

[4] Sahasakul Y, Panchavinnin P, Chaithiraphan S, Sakiyalak P. Echocardiographic diagnosis of a ruptured aneurysm of the sinus of Valsalva: operation without catheterisation in seven patients. Br Heart J 1990;64:195–198. 2206711 10.1136/hrt.64.3.195 PMC1024371

[5] Weinreich M, Yu PJ, Trost B. Sinus of Valsalva aneurysms: review of the literature and an update on management. Clin Cardiol 2015;38:185–189. 25757442 10.1002/clc.22359 PMC6711005

[6] Takach TJ, Reul GJ, Duncan JM, Cooley DA, Livesay JJ, Ott DA, et al Sinus of Valsalva aneurysm or fistula: management and outcome. Ann Thorac Surg 1999;68:1573–1577. 10585023 10.1016/s0003-4975(99)01045-0

