# Supplementary figures and images for: Ruptured sinus of Valsalva aneurysm mimicking infective endocarditis in a patient with Down syndrome: case report
Source: Eur Heart J Case Rep. 2026 Jul 9;10(7):ytag495. doi: 10.1093/ehjcr/ytag495 (PMC13395079; doi:10.1093/ehjcr/ytag495)

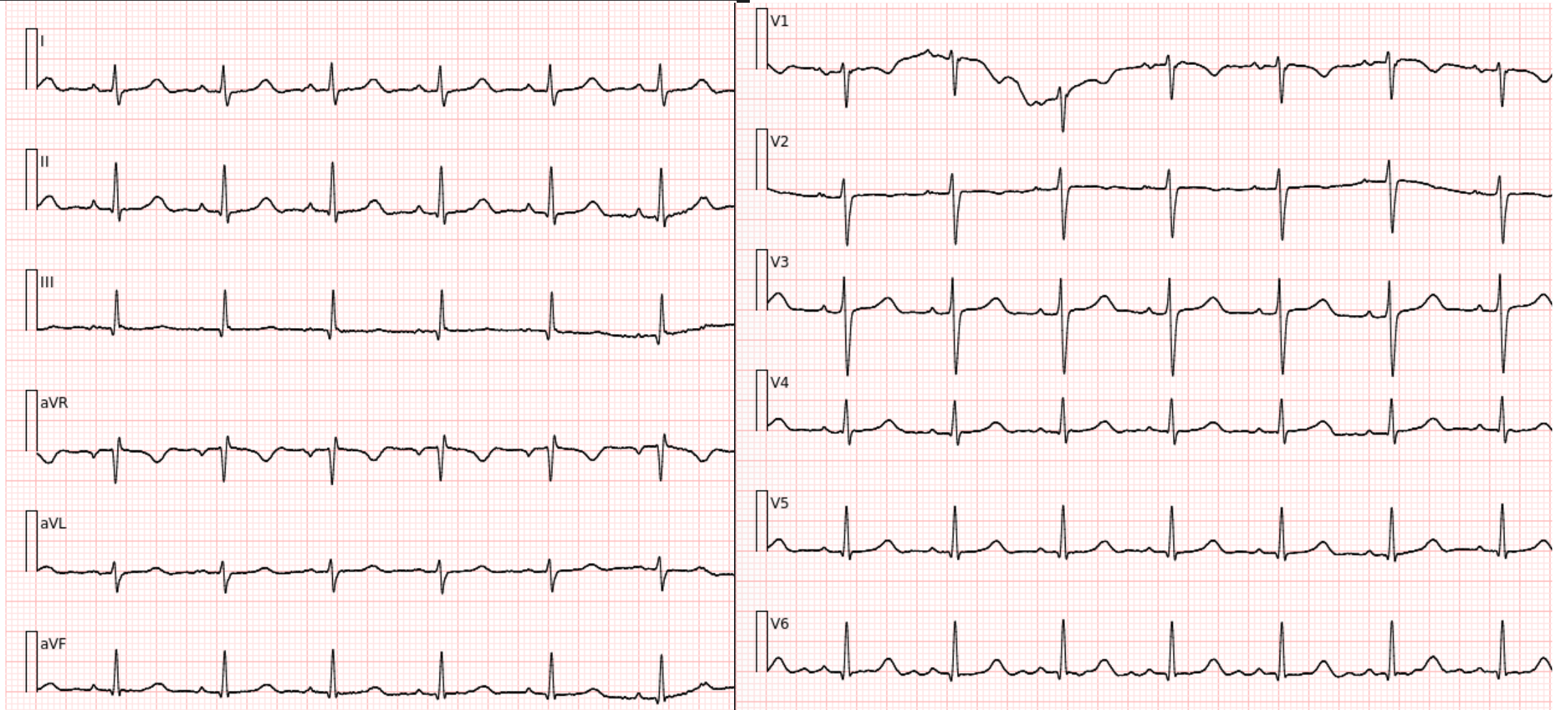

Supplement: ytag495_Supplementary_Data [file ytag495_supplementary_data.zip › Fig suppl 1 ECG.png]

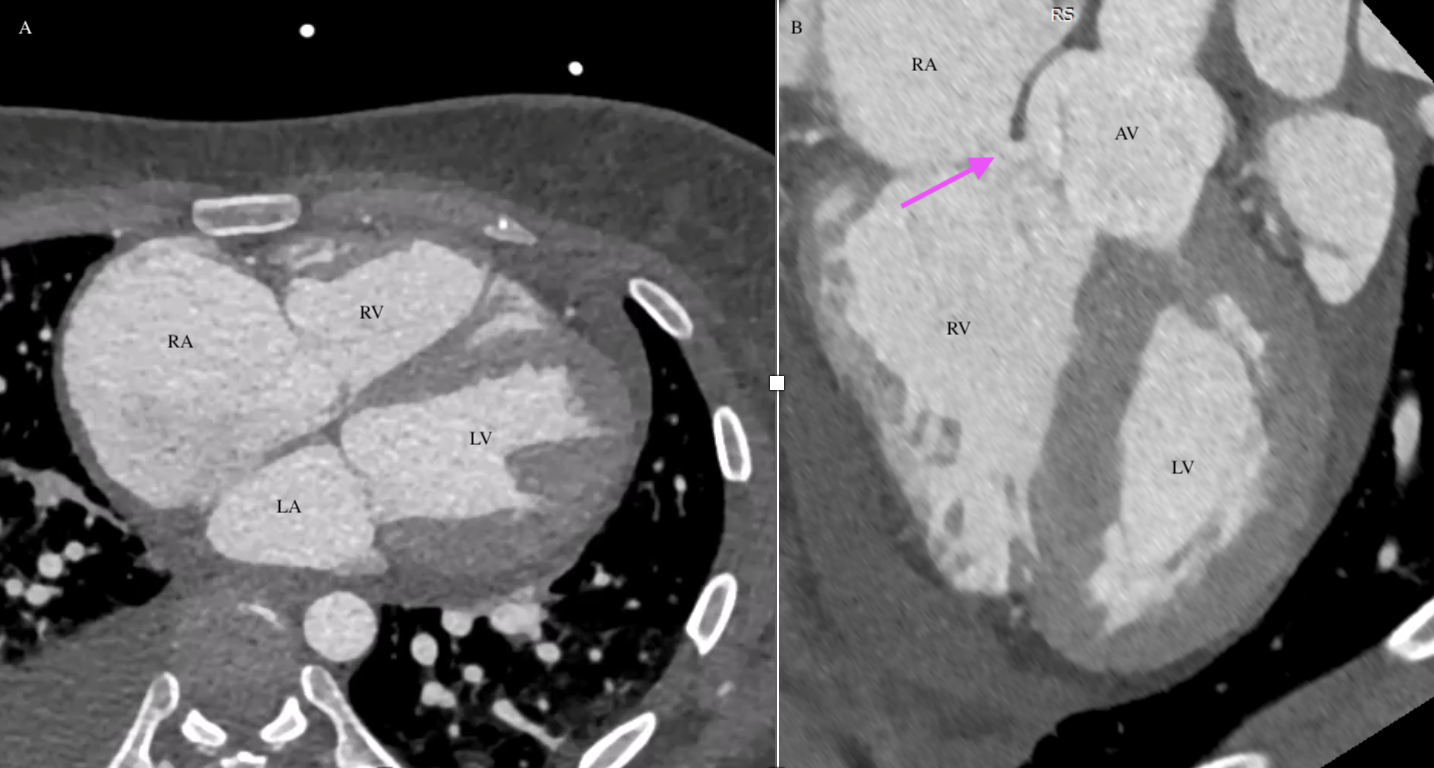

Supplement: ytag495_Supplementary_Data [file ytag495_supplementary_data.zip › Fig suppl 2 CT.png]
